# Exposure to the Paralytic Shellfish Toxin Producer *Alexandrium catenella* Increases the Susceptibility of the Oyster *Crassostrea gigas* to Pathogenic Vibrios

**DOI:** 10.3390/toxins8010024

**Published:** 2016-01-15

**Authors:** Celina Abi-Khalil, Carmen Lopez-Joven, Eric Abadie, Veronique Savar, Zouher Amzil, Mohamed Laabir, Jean-Luc Rolland

**Affiliations:** 1 Institut Français de Recherche pour l’Exploitation de la Mer (IFREMER), Interactions-Hôtes-Pathogènes-Environnements (IHPE UMR 5244), University of Perpignan Via Domitia, Centre National de la Recherche Scientifique (CNRS), University of Montpellier, Montpellier F-34095, France; celina.abi-khalil@umontpellier.fr (C.A.-K.); cljoven@gmail.com (C.L.-J.); 2 Center for MARine Biodiversity, Exploitation and Conservation (MARBEC, UMR 9190), University of Montpellier, Centre National de la Recherche Scientifique (CNRS), Institut de Recherche pour le Développement (IRD), Institut Français de Recherche pour l’Exploitation de la Mer (IFREMER), Montpellier F-34095, France; eric.abadie@ifremer.fr (E.A.); mohamed.laabir@univ-montp2.fr (M.L.); 3 Institut Français de Recherche pour l’Exploitation de la Mer (IFREMER), Phycotoxines Laboratory, l’Iled’Yeu street BP 21105, Nantes Cedex 3 F-44311, France; veronique.savar@ifremer.fr (V.S.); zouher.amzil@ifremer.fr (Z.A.)

**Keywords:** harmful algae, environment, interaction, pathogens, defense, paralytic shellfish toxin

## Abstract

The multifactorial etiology of massive *Crassostrea gigas* summer mortalities results from complex interactions between oysters, opportunistic pathogens and environmental factors. In a field survey conducted in 2014 in the Mediterranean Thau Lagoon (France), we evidenced that the development of the toxic dinoflagellate *Alexandrium catenella*, which produces paralytic shellfish toxins (PSTs), was concomitant with the accumulation of PSTs in oyster flesh and the occurrence of *C. gigas* mortalities. In order to investigate the possible role of toxic algae in this complex disease, we experimentally infected *C. gigas* oyster juveniles with *Vibrio tasmaniensis* strain LGP32, a strain associated with oyster summer mortalities, after oysters were exposed to *Alexandrium catenella*. Exposure of oysters to *A. catenella* significantly increased the susceptibility of oysters to *V. tasmaniensis* LGP32. On the contrary, exposure to the non-toxic dinoflagellate *Alexandrium tamarense* or to the haptophyte *Tisochrysis lutea* used as a foraging alga did not increase susceptibility to *V. tasmaniensis* LGP32. This study shows for the first time that *A. catenella* increases the susceptibility of *Crassostrea gigas* to pathogenic vibrios. Therefore, in addition to complex environmental factors explaining the mass mortalities of bivalve mollusks, feeding on neurotoxic dinoflagellates should now be considered as an environmental factor that potentially increases the severity of oyster mortality events.

## 1. Introduction

The mortality of the juvenile pacific oyster *Crassostrea gigas* is the result of complex interactions between oysters, their environment and opportunistic pathogens. It is a global phenomenon that has been observed several times in marine systems of different countries, including Brazil, the USA [1,2] and recently reported in New Zealand, Australia and Europe [3,4,5,6]. In France, high *C. gigas* mortality rates (60%–100%) have been reported since 1991. They are of major concern to oyster farmers [7,8] due to the important economic losses. Pathogenic populations of *Vibrio* from the Splendidus clade have been associated with the recurrent “summer mortality” outbreaks in *C. gigas* juveniles [9,10,11,12,13]. Among them is *Vibrio tasmaniensis* LGP32 [10], whose pathogenic processes have been characterized in detail [14,15,16]. The ostreid herpes virus OsHV-1 μVar was also associated with abnormal mortalities of *C. gigas* in France [17,18]. It is believed that abiotic factors enhance the susceptibility of oysters to infections by various pathogens, such as bacteria and viruses [9,10,19]. Temperature was actually found to be one major driving environmental factor influencing oyster mortality [20]. However, when considered individually, pathogens, temperature, reproductive effort, diet and farming practices donot account for the global increase in oyster mortalities in recent years [20,21,22,23].

*C. gigas* oysters, like other bivalve mollusks, are filter feeders that feed on micro-phytoplankton, including toxic dinoflagellates. Among these dinoflagellates, *Alexandrium catenella*, a paralytic shellfish toxin (PST) producer, is observed worldwide [24]. Extensive blooms of this species have been reported in various marine environments, such as the Pacific Ocean of North America [25], Chile [26], New Zealand [27], Japan, China, South Korea [28,29] and the western Mediterranean Sea [30,31]. In the Mediterranean Sea, blooms of *A. catenella* were observed in confined areas, such as lagoons and harbors [32,33]. Since 1998, several *A. catenella* blooms were observed in spring and/or autumn in the Mediterranean ThauLagoon (France, N 43°25′, E 03°39′), a shallow lagoon open to the sea and holding an important oyster (*C. gigas*) production (10,000 tones·year^−1^) [34,35,36,37,38]. In Thau Lagoon, *A. catenella* could reach high densities during bloom periods (3 × 10^6^–14 × 10^6^ cells·L^−1^) with toxin contamination in bivalves frequently exceeding the sanitary threshold over which the bivalves are considered dangerous for consumption [39].

It is now established that exposure of mollusks to high concentrations of PST-producing dinoflagellates could negatively affect the feeding, burrowing and survival of these bivalves [40,41,42,43]. Paralytic shellfish toxin (PST) producers are known to negatively affect the physiological and cellular processes of oysters, such as filtration and digestion [44,45,46,47]. Feeding on these neurotoxic dinoflagellates leads to an accumulation and biotransformation of PSTs mainly in the digestive gland [48,49]. Interestingly, these potent toxic algae impact the oyster immune system and induce an increase of reactive oxygen species (ROS) production and an inhibition of hemocyte phagocytosis [50,51,52]. Importantly, Medhioub *et al.* (2013) showed that the PST-producing *A. catenella* induces apoptosis in *C. gigas* hemocytes [53]. More recently, it has been shown that exposure to a toxic strain of *A. catenella* modifies the host-pathogen interaction by reducing the prevalence of OsHV-1 μvar infection [54]. However, the effects of *A. catenella* on the susceptibility of juvenile oysters to the bacterial pathogens involved in the summer mortality disease have not been investigated to date.

Here, we conducted a field study to investigate the relationship between the dynamics of *A. catenella* in the water column, the bioaccumulation of PSTs in oyster flesh and their mortality in the Thau Lagoon. The detection of PSTs in the flesh of oysters during a mortality event prompted us to investigate the negative effects of toxic algae on oyster susceptibility to pathogenic vibrios. In agreement with our field observations, controlled experiments showed that an exposure of *C. gigas* to *A. catenella* significantly impacts the survival of these mollusks when further infected with the pathogenic *V. tasmaniensis* strain LGP32.

## 2. Results

### 2.1. Occurrence of an Oyster Mortality Event during a Toxic Alexandrium catenella in the Thau Lagoon

**Figure 1:**
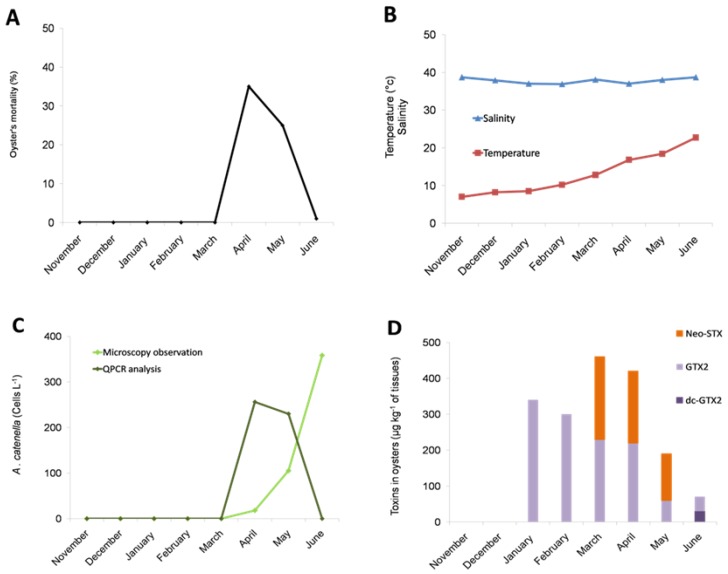
Field survey inside an oyster farm in Thau Lagoon in 2014. Oyster mortality (**A**); water temperature and salinity (**B**); abundance of *A. catenella* in water (**C**); accumulation of paralytic shellfish toxins in surviving oysters (**D**). The bar charts represent (in %) the temporal toxin in oysters. (Neo-STX for neosaxitoxin, GTX2 for gonyautoxin 2 and dc-GTX2 for decarbamoyl gonyautoxin 2).

We monitored the dynamics of toxic algae in the Thau in 2014, when a massive mortality of oyster juveniles occurred in April and May, as recorded in the French Shellfish Observatory Network database (RESCO for Réseau d'observations conchylicoles) (Figure 1A). The mortality event occurred when the seawater temperature was between 13 °C and 17 °C (Figure 1B). Interestingly, *A. catenella* cells were observed in the water column from the Thau Lagoon in April, May and June 2014 at concentrations of 18, 105 and 358 cells·L^−1^, respectively, when the mortality event occurred (Figure 1C). The identification of *A. catenella* DNA in April and May 2014 at concentrations corresponding to 256 and 230 cells·L^−1^, respectively, was confirmed by qPCR amplification (Figure 1C).

In addition to the presence of *A. catenella* in the water column, PSP toxins were detected in the flesh of juvenile oyster cultured in the Thau Lagoon. Importantly, neosaxitoxin (Neo-STX) was detected at high rates (up to 460 μg/kg of oyster flesh) in March, April and May 2014 concomitantly with the detection of *A. catenella* in the environment (Figure 1D). Interestingly, when mortality occurred (April–May 2014), the PSP rates tended to decrease in surviving oysters with no more Neo-STX being detected in June 2014, at the end of the mortality event. After the mortality event, in June 2014, neo-STX was no longer detected in surviving oysters; only low rates of gonyautoxin 2 (GTX2) and decarbamoyl gonyautoxin (dc-GTX2) were detected.

### 2.2. Exposure to Alexandrium catenella Increases Oyster Mortality

**Figure 2:**
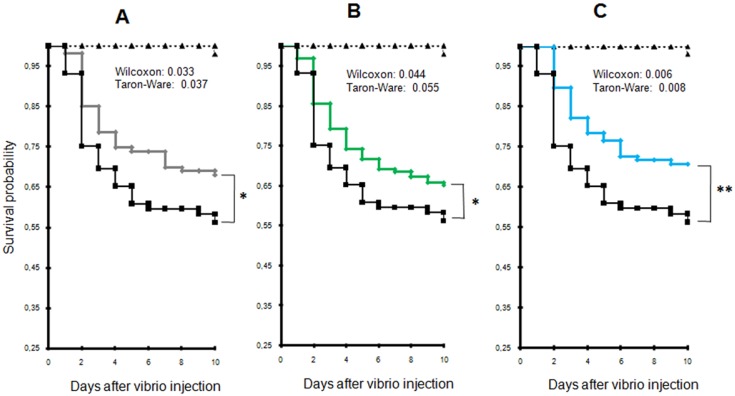
Comparison of Kaplan-Meier survival curves generated for oysters exposed to different algae and for starved mollusks. (**A**) Oysters fed *Alexandrium catenella versus* starved animals; (**B**) oysters fed *A. catenella versus* those fed *Alexandrium tamarense*; (**C**) oysters fed *A. catenella versus* those fed *Tisochrysis lutea*. *A. catenella* (black); *A. tamarense* (green); *T. lutea* (blue). Control oysters that were not infected by *Vibrio tasmaniensis* LGP32 or were injected with sterile seawater (SSW) are represented in black dashed triangles. * *p* < 0.05, ** *p* < 0.01.

To determine whether the presence of *Alexandrium catenella* could increase oyster mortality, juvenile oysters were exposed for 48 h either to the toxic strain of *A. catenella* (ACT03) or to *A. tamarense* (ATT07) or *T. lutea* used as foraging algae. Algal density was monitored in tanks all over the experiments showing similar concentrations in tanks during the time of experiment exposure (Figure S1). After a 24 h exposure to algae, oysters were injected with a lethal dose of *Vibrio tasmaniensis* LGP32 or sterile seawater (SSW), and oyster mortality was monitored for 10 days after their infection. We observed a significant increase in the mortality of oysters previously exposed to the toxic dinoflagellate *A. catenella* in comparison to individuals that were starved or exposed to *A. tamarense* or *T. lutea* (*p* = 0.033, 0.044 and 0.006, Wilcoxon test, respectively) (Figure 2). The LD_30_ (lethal dose 30%) was reached at Day 3 for animals previously fed with *A. catenella*, while it occurred at Days 7, 6 and 9 for oysters starved, fed with *A. tamarense* or fed with *T. lutea*, respectively. In other words, at Day 10, the mortality of oysters that were previously exposed to *A. catenella* was 35%, 27% and 53% higher than that of oysters unfed or fed with *A. tamarense* or *T. lutea*, respectively. In the absence of infectious challenge, no mortalities were recorded over 10 days for control animals injected with sterile sea water (SSW), for starved oysters and for oysters fed with *A. catenella*, *A. tamarense* or *T. lutea*.

### 2.3. PSTs Accumulate in Infected Oysters

**Figure 3:**
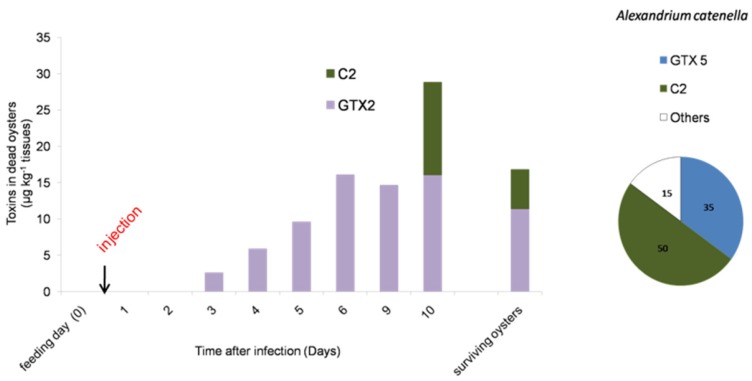
Evolution of the paralytic shellfish toxin content (μg·kg^−1^ wet weight of mollusk) in dead oysters during the *Vibrio tasmaniensis* LGP32 infection experiment. The bar charts represent (in %) the temporal toxin in oysters. The pie charts represent (in %) the toxin composition of *Alexandrium catenella*. (C2 for *N*-sulfocarbamoyltoxin 2, GTX2 for gonyautoxin 2 and GTX5 for gonyautoxin 5).

The *Alexandrium* sp. used in our experiment were controlled for their PST content. The ACT03 strain showed a toxin amount of 5.3 ± 0.4 pg toxins·cell^−1^. The specific toxicity of this strain was 1 ± 0.1 pg STX equivalent·cell^−1^. The ACT03 PST profile was characterized by the presence of carbamate, decarbamoyl and *N*-sulfocarbamoyltoxins. The following toxins were detected in decreasing concentrations: *N*-sulfocarbamoyltoxin 2 C2 (50%), GTX5 (35%), GTX4 (12%), GTX1 (1%) and Neo-STX (1%), with STX and dc-STX, GTX2, GTX3 and C4 present as trace amounts (Figure 3). No toxins were found in the *Alexandrium tamarense* strain (ATT07). In order to monitor the PST profile in oysters in our controlled experiment, dead oysters fed with *A. catenella* and infected with *V. tasmaniensis* were collected every day. PSTs were not detected in the most sensitive oysters, which died after one and two days of incubation. PSTs were detected in dead oysters after three days of incubation (Figure 3). Then, the toxicity level increased to reach 29 ± 7 μg·kg^−1^ tissue wet weight at Day 10. Moreover, the toxin profile displayed a substantial change in comparison to that of *A. catenella* cells (Figure 3). Among the toxins produced by *A. catenella*, only C2 was detected in oysters 10 days after the beginning of the experiment. GTX2, which was present as a trace in *A. catenella* cells, represented 100% of the total toxins measured in oysters that died at Days 3, 4, 5, 6 and 9 and was the major toxin compound (57%) at the end of the experiment (Day 10). Interestingly, the toxicity level reached 17 ± 5 μg·kg^−1^ tissue wet weight in oysters considered as survivors 12 days after the infection (Figure 3).

## 3. Discussion

Results from our laboratory experiments demonstrated that exposure to the neurotoxic *Alexandrium catenella* increases the susceptibility of *Crassostrea gigas* oysters to the pathogenic *V. tasmaniensis* LGP32. Indeed, a significant increase in oyster mortality induced by *Vibrio* infection was observed after exposure to *A. catenella.* Interestingly, we earlier showed that this toxic dinoflagellate induces the apoptosis of *C. gigas* hemocytes (immune cells) at PST concentrations similar to those generally observed *in situ* during a bloom of toxic algae [53]. Apoptosis was observed 24 h after exposure to *A. catenella*, which corresponds to the time we used for oyster infection in our experiment. Such destruction of the hemocytes, which play a key role in the defense mechanism of invertebrates [55], could strongly affect the capacity of *C. gigas* to resist bacterial infection and be responsible for the increased susceptibility of oysters observed here. Contrary to our results, exposure to *A. catenella* was shown to reduce the infection of oysters by the OsHV-1 herpesvirus [54]. The different susceptibility of oysters to those different pathogens is probably due to different immune reactions required to control bacterial and viral pathogens [56]. It can indeed be hypothesized that PSTs polarize the oyster immune response by triggering immune mechanisms that predispose to bacterial infections, but protect from viral infections. However, the potential lytic activity of the ACT03 strain towards hemocytes due to allelochemical compounds could not be ruled out and must be considered as an additional factor increasing the sensitivity of oysters to pathogens [57].

Supporting our hypothesis of a negative effect of toxic algae on oyster immunity, we observed an accumulation of toxins in the flesh of oyster batches showing higher mortality rates. Interestingly, no PSTs were found during the first and the second day of the incubation in the tissues of dead oysters that were fed with *A. catenella* and infected with *V. tasmaniensis*. GTX2, which was absent in the ACT03 cells, appeared in dead oysters three days after infection, and its concentration increased continuously until the 10th day of experimental exposure and during the *in situ* exposure. Data showed differences between toxins in the ingested *A. catenella* cells and those found in fed oysters. This could be attributed to a selective uptake of the toxins in oyster tissues, metabolic interconversion and elimination of the toxins [47,58,59,60]. Several studies suggested that an active biotransformation/interconversion of PSTs occurred [58,61]. The toxins that were accumulated in oysters in the experimental infection or in oyster individuals exposed *in situ* to natural *A. catenella* blooms can result from the transformation of some toxins (GTX3, C2 and others) produced by this dinoflagellate. This suggests that an important biotransformation process occurred in the *C. gigas* tissues. In our experiments, C2 and GTX2 accumulated in surviving oysters (Figure 3). We can speculate that these toxins, resulting from biotransformation, may have low toxicity and presumably did not affect the capacity of oysters to resist vibrio infection.

One important finding from this study is that *C. gigas* mortality observed in 2014 occurred while oyster flesh was contaminated by PSTs. These results correspond to previous works showing that PSTs were present at concentrations as high as 920 μg·kg^−1^ of digestive gland wet weight in oysters exposed to toxic algae in Thau Lagoon in May 2011 [62] when oyster mortality also occurred (April–May 2011 according to the French Shellfish Observatory Network). In the present study, toxins had been detected in oyster tissues since January 2014, while mortalities occurred only in April and May 2014. In agreement with our experimental data, this indicates that oyster intoxication by PSTs alone is not sufficient to induce mortalities, but could rather participate in the induction of oyster mortality in a multifactorial way that involves vibrios and likely other pathogens [13,63].

This study showed that *A. catenella* increases the susceptibility of *C. gigas* oysters to pathogenic *V. tasmaniensis*. To our knowledge, this is the first time that a direct link between a previous exposure of marine invertebrates to a paralytic shellfish toxin (PST) producer and the mortality induced by a pathogenic microorganism has been established. The interaction *A. catenella/C. gigas/Vibrio* was supported by the co-occurrence of oyster mortality and PST presence in oyster organs. The field observation suggested that *A. catenella* development *in situ* could trigger *C. gigas* mortality. Among the dinoflagellates producing PSTs, the genus *Alexandrium* is one of the major genera with respect to its diversity, magnitude and consequences on the ecosystem components [24]. Studies will provide further insight into the relationships between the established and emerging toxic dinoflagellates and mass mortalities of marine invertebrates. In particular the role of these algae in predisposing marine animals to microbes (bacteria and/or virus) occurring frequently in the environment should be the subject of further study. Other research focusing on direct interactions between toxins and bacteria would be required to better understand the mechanisms involved in these interactions. To conclude, in addition to complex environmental factors explaining mass mortalities of bivalve mollusks, feeding on toxic algae should now be considered a contributing factor.

## 4. Experimental Section

### 4.1. Field Survey in Thau Lagoon

#### 4.1.1. Collection of Environmental Samples and Processing

**Figure 4:**
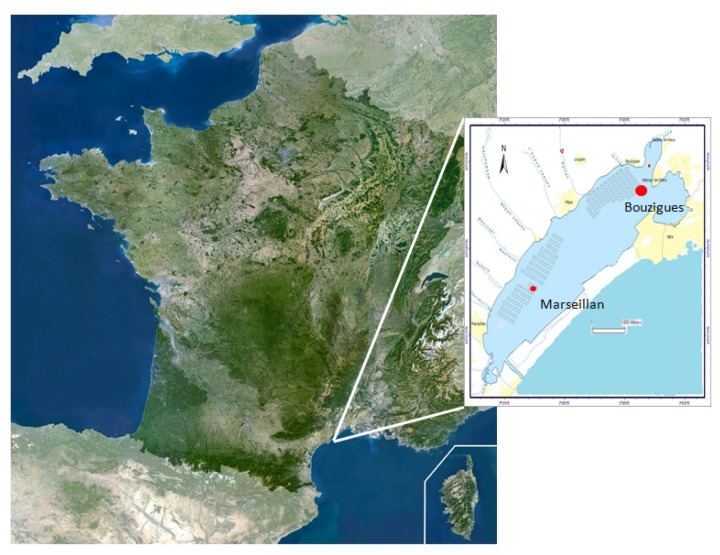
Map showing locations of the sampling stations in Thau Lagoon.

Phytoplankton samples from Thau Lagoon (Languedoc-Roussillon, France) were collected each month from November 2013–June 2014 inside the oyster farm named Bouzigues (N 43°26.058′ and E 003°39.878′) (Figure 4). Sea water (duplicate) was collected from the sub-surface (−50 cm) using a pump. On the boat, 20–30 liters of water were concentrated through 20-μm pore filters to a final volume of 50 mL (fraction >20 μm). Total DNA from 1 mL of this fraction was extracted and purified according to the standard procedure of the current protocols in molecular biology, Unit 2.2.1 [64]. After purification, DNA was conserved in 100 μL of water at −20 °C until PCR was performed.

#### 4.1.2. Dynamics of *Alexandrium catenella*

Species belonging to the *Alexandrium tamarense* complex are often difficult to separate. This complex is composed of three morphospecies, namely *A. fundyense* (Balech), *Alexandrium catenella* (Whedon & Kofoid) Balech and *A. tamarense* (Lebour) Balech. Taxa, such as *A. catenella* and *A. tamarense*, are considered as cryptic species [65]. Recently, John *et al.* [66] proposed to rename *A. catenella* (Whedon & Kofoid) Balech *Alexandrium pacificum* (Litaker). However, this proposition is still under debate [67]. In Thau Lagoon, Genovesi *et al.* [36] showed the simultaneous presence of the toxic *A. catenella* and the non-toxic *A. tamarense* [68] that belong to Group IV and Group III, respectively. By microscopic observation, *A. catenella* cannot be formally identified; molecular identification is therefore the most accurate method to identify this species accurately in a preserved natural samples.

From field-preserved Thau water samples, *Alexandrium catenella/tamarense* abundance (cells·L^−1^) was determined using photonic microscopy counting. As specified above, based on the morphological characteristics, it was difficult to discriminate *A. catenella* from *A. tamarense* in the field samples. To specifically identify and quantify the presence of *A. catenella* in water, we performed a quantitative method using the primers FACAT28 and RACATAM269 from Genovesi *et al.* [36] that target specifically the 18S–28S rRNA ITS region of *A. catenella*. Briefly, PCR amplifications were performed in the Light Cycler 480 (Roche Diagnostics GmbH, Mannheim, Germany). In short, the following components were mixed: 1 μM of each primer (3.33 μM) and 3 μL of reaction mix (Light Cycler^®^ 480 SYBR^®^ Green I Master mix, Roche Diagnostics GmbH, Mannheim, Germany) in a final volume of 5 μL. DNA (1 μL) was added as the PCR template to the mix, and the following run protocol was used: initial denaturation at 95 °C for 5 min; 95 °C for 10 s; 58 °C for 10 s; 72 °C for 10 s. A subsequent melting temperature curve was performed between 65 °C and 97 °C with a heating rate of 0.11 °C/s, a continuous fluorescence measurement and a cooling step to 40 °C. Each PCR was performed in duplicate. To determine the qPCR efficiency, the standard curve was generated using five serial dilutions (1:1, 1:3, 1:7, 1:15, 1:31) of a DNA sample of the *A. catenella* ACT03 strain. Before quantitative PCR analysis, a significant linear relationship (*R*^2^ = 0.99) was demonstrated between the cycle threshold (Ct) and the logarithm of the initial concentration of *A. catenella* ACT03 from 4 to 4000 cells·L^−1^ (the detection limit). Moreover, the PCR product analysis on agarose gel and by melting curve revealed a unique lane (240 bp) and a unique peak (80 °C), respectively, indicating the formation of a single PCR product with no artifacts (data not shown).

#### 4.1.3. Oyster Mortality

Oyster mortality was monitored in Thau Lagoon from November 2013–June 2014 by the French Shellfish Observatory Network [69]. Oysters were natural diploid *Crassostrea gigas* juveniles collected in 2012 and 2013 in the basin of Arcachon, France (initial average shell length ≤30 mm,). Oysters were placed inside an oyster farm, called Marseillan East (N 43°36.461′ and E 003°55.394′, Marseillan, Languedoc-Roussillon, France), that corresponds to a representative site of oyster mortalities that occur throughout the lagoon (Figure 4). In fact, mortalities were shown to affect different oyster farms within Thau Lagoon (in Marseillan and Bouzigues) identically in terms of kinetics and magnitude [23].

#### 4.1.4. Oyster Biotoxin Contamination

To monitor contamination by PSTs, juvenile oysters (Batch 2013.2) produced at the Institut Français de Recherche pour l'Exploitation de la Mer (IFREMER) Hatchery in Bouin, France (initial average shell length 30 ± 0.4 mm), were immersed in ThauLagoon at the oyster farm named Bouzygues (N 43°26.058′ and E 003°39.878′) in November 2013 (Figure 4). Five juvenile oysters were randomly collected each month from November 2013–June 2014. Pooled tissues of oysters were frozen at −20 °C until toxin extraction and subsequent analyzes were performed.

### 4.2. Experimental Infections

#### 4.2.1. Oysters

Oysters used for experimental infections (Batches 2013.2 and 2014.2) were diploid *Crassostrea gigas* juveniles produced at the IFREMER Hatchery in Bouin, France (initial average shell length 30 ± 0.4 mm).

#### 4.2.2. Algae Production

The experiments were carried out with a toxic strain of *Alexandrium catenella* (ACT03), a non-PST producing strain, *Alexandrium tamarense* (ATT07), and the haptophyte, *Tisochrysis lutea*, which is considered a foraging alga. ACT03 and ATT07 were isolated from Thau Lagoon in 2003 and 2007, respectively. Examination of the morphology of the studied strain ACT03 clearly showed the absence of the ventral pore between the plates 1’ and 4’ and the ability to form chains of up to eight cells during the exponential growth phase. This corresponds to the description of *Alexandrium catenella* (Whedon & Kof) Balech. In contrast, cells from the ATT07 culture showed clearly the presence of this previously-described ventral pore, and these are always observed in single or paired cells. Analysis of the nuclear rRNA fragment, including ITS1, the 5.8S rRNA gene, ITS2, and the D1/D2 28S rRNA genes revealed that the strain ACT03 belonged to the *A. catenella* species from Group IV (temperate Asian clade) and that the strain ATT07 belongs to *A. tamarense* (Group III) [36]. Dinoflagellates were grown at 20 °C under cool-white fluorescent illumination (100 μmoles photons/m^2^/s) in batch cultures, using a 12 h:12 h light:dark cycle. The enriched natural sea water (ENSW) culture medium was carried out following Harrison *et al.* [70] and was characterized by a salinity of 35. For the experiments, algae populations were in their exponential growth phase.

#### 4.2.3. Pathogenic Bacterial Strain

Bacteria for infection were prepared as follows. A colony of *Vibrio tasmaniensis* LGP32 wild-type previously isolated on a Zobell media Petri dish was grown overnight at 20 °C in liquid medium under agitation (150 rpm). Cells were then washed three times with sterile seawater (SSW) by centrifugation (10 min, 1000 g, 20 °C).

#### 4.2.4. Experimental Design

**Figure 5:**
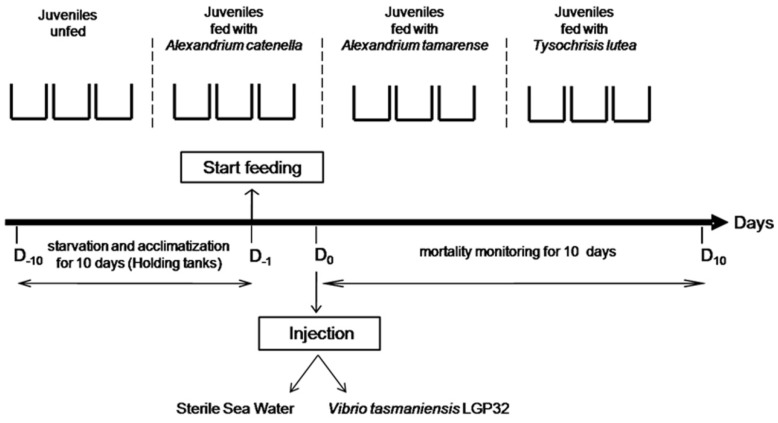
Laboratory experimental design.

Two independent experimental exposures to *Alexandrium catenella* were carried out according to the protocol described by Rolland *et al.* [47]. Briefly, after 10 days of acclimatization without feeding, *Crassostrea gigas* juveniles were randomly placed into tanks (50 individuals per tank, 3 tanks per condition) containing 6 liters of filtered (0.2 μm) seawater thermo-regulated at 22 °C. Then, oysters were subjected to different treatments, including exposure for 24 h to the toxic *A. catenella* and the non-toxic *Alexandrium tamarense* or *Tisochrysis lutea* at an initial concentration of 2 × 10^6^ cells·L^−1^, similar to the conditions observed during a natural bloom in Thau Lagoon [38]. Over the experiment, the algae concentration was maintained between 1 × 10^6^ and 3 × 10^6^ cells·L^−1^ by adding algae to the sea water tanks. In control tanks, no algae were added. After a 24 h exposure, each batch of oysters received a 30% lethal dose (LD_30_) of bacteria determined at Day 7. For that, each juvenile was injected with one dose of *V. tasmaniensis* LGP32 (50 μL/animal; 2 × 10^8^ and 1 × 10^9^ for Batches 2013.2 and 2014.2, respectively) into its adductor muscle. Control oysters were injected with 50 μL of sterile seawater (SSW). The mortality of juvenile oysters was monitored daily over 10 days (Figure 5). To determine the level of PST contamination of oysters, dead animals were collected each day for the duration of the experiment. Pooled tissues were frozen at −20 °C until the extraction of the toxin was carried out.

### 4.3. Neurotoxins Analysis

To extract the PSTs, 5 mL of 0.1 N hydrochloric acid were added, and the samples were mixed with a high-speed homogenizer (15,000 rpm) for 2 min. The pH was adjusted between 2.0 and 4.0, then the samples were centrifuged at 4200 g for 10 min at 4 °C. The supernatants were filtered on 10-kDa polyethersulfone (PES) filters, and the toxin content was analyzed using the liquid chromatography with fluorescence detection (LC/FD) PSP toxin analyses method of Van De Riet [71]. The toxins GTXs, dc-GTXs, dc-STXs and STXs were separated using a reverse chromatography column (Zorbax Bonus RP, 3.5 μM, 4.6 × 150 mm, Agilent Technologies, Massy, France) with a flow rate of 0.8 mL·min^−1^. The eluent pH and/or column temperature were optimized to separate dc-GTX3/GTX5/dc-GTX-2 and C1/C2. The toxin concentrations were determined using certified standards provided by CNRC (Halifax, NS, Canada).

### 4.4. Statistical Analysis

The non-parametric Kaplan-Meier test was used to estimate Wilcoxon and Tarone-Ware values for comparing the experimental survival curves [72]. A confidence limit of 95% was used to test the significance of differences between groups. Data for the same experiment groups were pooled since there were no significant differences (*p* > 0.05). Values are the mean ± SD from two independent experiments * *p* < 0.05, ** *p* < 0.01, *** *p* < 0.001.

## Supplementary Materials

The following figure is available online at www.mdpi.com/2072-6651/8/1/24/s1. Figure S1. Variation of cell concentration in tanks during the experiments. *Alexandrium catenella* (blue); *Alexandrium tamarense* (red); *Tisochrysis lutea* (green).
