# Supplementary material for: Exposure to the Paralytic Shellfish Toxin Producer Alexandrium catenella Increases the Susceptibility of the Oyster Crassostrea gigas to Pathogenic Vibrios
Source: Toxins (Basel). 2016 Jan 15;8(1):24. doi: 10.3390/toxins8010024 (PMC4728546; doi:10.3390/toxins8010024)
Supplement: Supplementary file 1 [file toxins-08-00024-s001.pdf]

Supplementary Materials: Exposure to the Paralytic Shellfish Toxins (PSTs) Producer *Alexandrium catenella* Increases the Susceptibility of the Oyster *Crassostrea gigas* to Pathogenic Vibrios

Celina Abi-Khalil, Carmen Lopez-Joven, Eric Abadie, Veronique Savar, Zouher Amzil, Mohamed Laabir and Jean-Luc Rolland

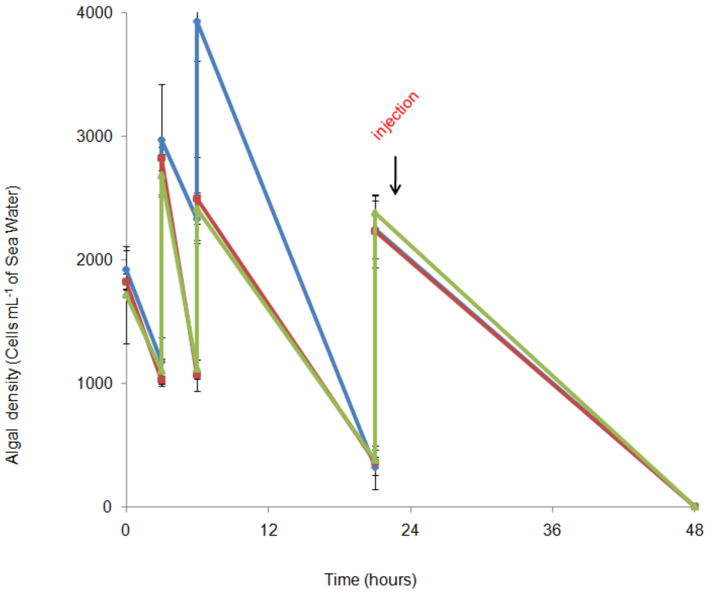

Figure S1. Variation of cell concentration in tanks during the experiments. *Alexandrium catenella* (Blue); *Alexandrium tamarense* (Red); *Tysochrisis lutea* (Green).
